# Autonomic characteristics of periodic limb movements: comparison of whole-night and stage N2 linear and non-linear heart rate variability

**DOI:** 10.1007/s10286-025-01184-y

**Published:** 2026-01-13

**Authors:** Elif Simin Issı, Selahattin Ayas, Elif Göksu Yiğit Tekkanat

**Affiliations:** 1 Department of Neurology, Faculty of Medicine, Afyonkarahisar Health Sciences University, Afyonkarahisar, Türkiye; 2 Department of Clinical Neurophysiology, Eskişehir City Hospital, Eskişehir, Türkiye; 3 Department of Algology, Faculty of Medicine, Eskişehir Osmangazi University, Eskişehir, Türkiye

**Keywords:** Periodic limb movement disorder, Autonomic nervous system, Heart rate variability, Sleep stage N2, Nonlinear dynamics, Cardiovascular risk

## Abstract

**Purpose:**

This study aimed to investigate autonomic alterations associated with periodic limb movements during sleep (PLMS) by comparing linear and non-linear heart rate variability (HRV) parameters across whole-night recordings and stage N2 non-rapid eye movement (NREM) sleep (N2).

**Methods:**

From 8082 polysomnographic (PSG) recordings, we identified 21 patients with PLMS and 28 age- and sex-matched controls. Linear and non-linear HRV indices were analyzed for whole-night recordings and the longest N2 segment. Periodic limb movement (PLM) indices and arousal-related parameters were also evaluated.

**Results:**

Compared with controls, patients with PLMS showed significantly higher standard deviation of normal-to-normal intervals (SDNN) and root mean square of successive differences (RMSSD), increased low-frequency (LF) power, and reduced approximate entropy (ApEn) and sample entropy (SampEn) across the whole night, together with a lower Stress Index (SI). No significant group differences were observed in high-frequency (HF) power or in the composite sympathetic/parasympathetic nervous system (SNS/PNS) indices. During stage N2, the PLM group exhibited significantly greater SDNN, Poincaré plot long-axis standard deviation (SD2), detrended fluctuation analysis alpha-1 exponent (DFA α1), and very-low-frequency (VLF) power, along with lower ApEn values. A significant increase in the SD2/SD1 ratio was also observed specifically during N2, whereas the LF/HF ratio showed only a non-significant upward trend. PLMS counts, indices, and arousal-related parameters were markedly elevated during stage N2.

**Conclusions:**

PLMS are characterized by increased autonomic variability (SDNN, SD2, DFA α1) but reduced complexity (ApEn, SampEn), particularly during stage N2. Stage-specific HRV assessment may provide novel insights into the cardiovascular implications of PLMS.

## Introduction

Periodic limb movements of sleep (PLMS) are characterized by repetitive extensor muscle contractions occurring at intervals of approximately 20–40 s during sleep, and are classified as a parasomnia that disrupts sleep microstructure and autonomic regulation. Polysomnography-based data report a prevalence of 4–11% in the adult population, with cases presenting either in association with restless legs syndrome or as isolated PLMS [1]. Consecutive muscle activations have been linked to transient increases in heart rate, elevations in blood pressure, and cortical arousals; over the long term, these alterations may contribute to an increased risk of hypertension, arrhythmias, and other cardiovascular events [2, 3]. Recent reviews have emphasized their potential role as a sleep-related cardiovascular risk factor [4, 5].

Heart rate variability (HRV) is a non-invasive method used to evaluate sympathetic–parasympathetic interactions. Beyond conventional time-domain (e.g., standard deviation of NN intervals (SDNN), root mean square of successive differences (RMSSD)) and frequency-domain parameters (e.g., low frequency (LF), high frequency (HF), and LF/HF ratio), non-linear metrics such as sample entropy (SampEn), detrended fluctuation analysis (DFA α1/DFA α2), and Poincaré plot indices (standard deviation 1 (SD1), standard deviation 2 (SD2), and SD2/SD1 ratio) provide a more detailed representation of the complexity and fractal properties of autonomic regulation [6, 7].

Event-locked studies, such as those by Sforza et al. (2005) and Sasai et al. (2013), consistently reported a transient sympathetic predominance (↑LF, ↑LF/HF) around limb-movement onset [8, 9]. More recent investigations have described increased HF-HRV and blood-pressure elevations during longer PLMS episodes (> 2.1 s), suggesting a time-dependent autonomic co-activation pattern [4, 10–12]. These complementary results indicate that PLMS may perturb autonomic balance through complex, duration-related interactions between sympathetic and parasympathetic systems. Taken together, these studies have substantially advanced the understanding of short-term autonomic dynamics associated with PLMS; however, most prior work has focused on event-locked segments or whole-night averages, providing only limited insight into stage-specific autonomic profiles.

Event-related studies have demonstrated sympathetic activation during the “pre-arousal” period, with increases in low-frequency (LF) and very-low-frequency (VLF) power approximately 1 min prior to the onset of periodic limb movements (PLMs) [8]. However, these clip-based approaches have not systematically evaluated stage N2, which constitutes nearly 50% of total sleep time and is known to cluster cardiovascular events. Because stage N2 constitutes roughly half of total sleep and coincides with the peak incidence of nocturnal arrhythmias, evaluating PLMS within this stage may clarify the mechanistic links between microarousals and cardiovascular stress [13].

Recent methodological evidence has confirmed the reproducibility of both linear and non-linear HRV indices across sleep stages, thereby supporting the reliability of stage-specific analyses [11].

Despite these insights, no previous study has directly compared whole-night and N2-restricted HRV—integrating both conventional and nonlinear indices—in adult PLMS. This study was therefore designed to address this gap. We hypothesized that stage N2 would exhibit the greatest autonomic disruption, with PLMS showing increased variability (SDNN, LF, DFA α1) but decreased complexity (ApEn, SampEn) relative to controls.

## Materials and methods

### Study design and participants

This retrospective study evaluated 8082 full-night polysomnography (PSG) recordings performed between January 2021 and December 2024 at the Eskişehir City Hospital Sleep Laboratory. Based on the periodic limb movement index (PLMI), two groups were formed: the “PLM group” (*n* = 21) included subjects with PLMI ≥ 15, and the age- and sex-matched “control group” (*n* = 28) included those with PLMI < 5. Exclusion criteria were atrial fibrillation, diabetes mellitus, chronic kidney disease, use of β-blockers, anticholinergic or dopaminergic medications, presence of psychiatric comorbidities, and apnea–hypopnea index (AHI) ≥ 10, diagnosed neurodegenerative disorders (e.g., Parkinson’s disease, multiple system atrophy, dementia with Lewy bodies), use of sedative-hypnotic medications, excessive alcohol or caffeine consumption, and all participants were medication-free for at least 48 h before PSG and had no acute medical or neurological conditions at the time of recording. The study was conducted in accordance with the Declaration of Helsinki and approved by the local ethics committee (Approval No. ESH/BAEK 2024/23).

### Polysomnographic data acquisition

All PSG data were collected using the Philips Sleepware G3 system (Philips Respironics, Eindhoven, Netherlands) with a sampling rate of 256 Hz. The standard montage included electroencephalogram (EEG: F3-M2, C3-M2, O1-M2), electrooculogram (EOG), submental and anterior tibialis electromyogram (EMG), single-lead chest electrocardiogram (ECG), thoracic and abdominal respiratory inductance plethysmography (RIP), oro-nasal airflow pressure transducer, and peripheral oxygen saturation (SpO_2_) via pulse oximetry. In nine earlier recordings with EMG channels sampled at 128 Hz, resampling to 256 Hz was performed prior to analysis to standardize the dataset.

### Sleep scoring and PLM assessment

Sleep staging was performed manually by two independent scorers on the basis of the American Academy of Sleep Medicine (AASM) criteria version 3.1, using 30-s epochs. Discrepancies were resolved through consensus. Periodic limb movements (PLMs) were defined as at least four consecutive EMG bursts in the anterior tibialis muscle, each lasting 0.5–10 s and occurring at intervals of 5–90 s. The PLM index (PLMI) was calculated as the number of PLMs per hour of total sleep time. PLMs temporally associated with EEG arousals (within ± 0.5 s) were classified as “PLM-arousal” events.

### RR signal processing and HRV computation

RR intervals derived from the ECG recordings were imported into the Kubios HRV Scientific Lite software (version 4.1.2.1; Kubios Oy, Kuopio, Finland). The default preprocessing settings of the software were applied as follows:

- Artifact correction: Performed using the Kubios algorithm with a low threshold setting.
- Detrending method: Smoothness priors (*λ* = 500).
- Interpolation: Equidistant interpolation at 4 Hz.
- Recordings with an artifact correction rate exceeding 5% were excluded from the analysis.
- Heart rate variability (HRV) parameters were computed automatically by Kubios and included the following domains:
- Time-domain parameters: Mean RR, mean HR, standard deviation of NN intervals (SDNN), root mean square of successive differences (RMSSD), the proportion of successive intervals differing by more than 50 ms (pNN50), HRV Triangular Index, and Stress Index.
- Frequency-domain parameters: Total power (0.003–0.40 Hz), very low frequency (VLF: 0.003–0.04 Hz), low frequency (LF: 0.04–0.15 Hz), high frequency (HF: 0.15–0.40 Hz), and the LF/HF ratio calculated using the Lomb–Scargle periodogram.
- Non-linear parameters: SD1, SD2, SD2/SD1 ratio, approximate entropy, sample entropy, and detrended fluctuation analysis (DFA α1/DFA α2).
- Autonomic nervous system indices: Parasympathetic nervous system (PNS) index and sympathetic nervous system (SNS) index.

### Segment selection

- Whole-night segment: The entire sleep duration between the “lights-off” and “lights-on” markers.

- Longest N2 segment: The longest continuous N2 sleep episode lasting ≥ 20 min.

### PLM metrics

For both segments, the following PLM metrics were calculated: total number of PLMs, periodic limb movement index (PLMI), number of PLMs associated with cortical arousals (PLM-arousals), and the PLM-arousal index (events per hour).

### Statistical analysis

Normality of data distribution was assessed using the Shapiro–Wilk test. Continuous variables with normal distribution are presented as mean ± standard deviation (SD) and were compared using the independent samples Student’s *t* test. Non-normally distributed variables are presented as median (interquartile range, IQR) and were compared using the Mann–Whitney *U* test. Categorical variables were analyzed using Pearson’s chi-square test. Groups were age- and sex-matched, and no significant differences were observed in body mass index (BMI). As potential confounders were already controlled by study design (apnea–hypopnea index [AHI] ≥ 10 was an exclusion criterion), no further covariate adjustment was necessary. Between-group comparisons of HRV parameters were therefore conducted using unadjusted *t* tests or Mann–Whitney *U* tests as appropriate. Effect sizes were calculated and reported as Cohen’s *d* (small, *d* < 0.50; medium, 0.50–0.79; large, ≥ 0.80), together with their 95% confidence intervals. All tests were two-tailed, and a *p* value < 0.05 was considered statistically significant.

## Results

A total of 21 patients with PLMs (PLM group) and 28 age- and sex-matched controls (control group) were included in the study.

### Demographic and clinical characteristics

No significant differences were observed between the groups in terms of age, sex, body mass index, smoking status, or Epworth Sleepiness Scale scores. Sleep efficiency was lower and the wake after sleep onset (WASO) percentage was higher in the PLM group. Additionally, both the apnea–hypopnea index (AHI) and oxygen desaturation index were significantly elevated in this group. As expected, the PLM index was markedly increased in the patient group (Table 1).

**Table 1:** Demographic and clinical (polysomnographic) characteristics of the study groups

Variable	Patients with PLM (*n* = 21)	Control group (*n* = 28)	*p*
Age (years), mean ± SD	43.6 ± 10.2	40.1 ± 7.3	0.169^a^
Gender, *n* (%)			0.862^b^
Female	14 (66.7%)	18 (64.3%)	
Male	7 (33.3%)	10 (35.7%)	
Height (cm), median (IQR)	165 (16)	165 (9.3)	0.738^c^
Weight (kg), median (IQR)	73 (16)	75 (19.3)	0.363^c^
Body mass index (kg/m^2^), mean ± SD	25.9 ± 4.6	27.6 ± 4.6	0.200^a^
Smoking, *n* (%)	9 (42.9%)	10 (35.7%)	0.612^b^
Epworth Sleepiness Scale, mean ± SD	5.7 ± 3.1	7.4 ± 3.1	0.059^a^
Total sleep time (min), median (IQR)	376 (40.8)	386.3 (31.8)	0.090^c^
Sleep efficiency (%), median (IQR)	91.5 (9.6)	94.4 (4.5)	0.016^c^
Sleep latency (min), median (IQR)	7.5 (15.7)	4.2 (10.5)	0.092^c^
REM latency (min), mean ± SD	145.8 ± 91.2	137.9 ± 71.2	0.736^a^
WASO (%), median (IQR)	8.5 (9.5)	5.6 (4.5)	0.044^c^
N1 (%), median (IQR)	3.2 (4.2)	2.9 (4.9)	0.649^c^
N2 (%), median (IQR)	61 (24)	57.5 (15.2)	0.551^c^
N3 (%), mean ± SD	15.6 ± 10.5	17.9 ± 8.9	0.417^a^
REM (%), mean ± SD	10.6 ± 7.0	14.7 ± 7.7	0.066^a^
Apnea–hypopnea index (events/h), mean ± SD	5.1 ± 1.9	3.1 ± 1.3	0.000^a^
REM AHI (events/h), median (IQR)	4.2 (7.3)	4.1 (4.7)	0.848^c^
NREM AHI (events/h), mean ± SD	4.9 ± 1.9	2.7 ± 1.3	0.000^a^
Oxygen desaturation index (events/h), mean ± SD	4.2 ± 2.9	2.6 ± 1.3	0.023^a^
PLM index (events/h), median (IQR)	23.5 (15.4)	0 (0.8)	0.000^c^

*SD* standard deviation, *M* median, *IQR* interquartile range, *PLM* periodic limb movements, *WASO* wake after sleep onset, *REM* rapid eye movement, *NREM* non-REM

^a^Student’s *t* test; ^b^Pearson Chi-Square Test; ^c^Mann–Whitney *U* Test

### Whole-Night HRV analysis

**Fig. 1:**
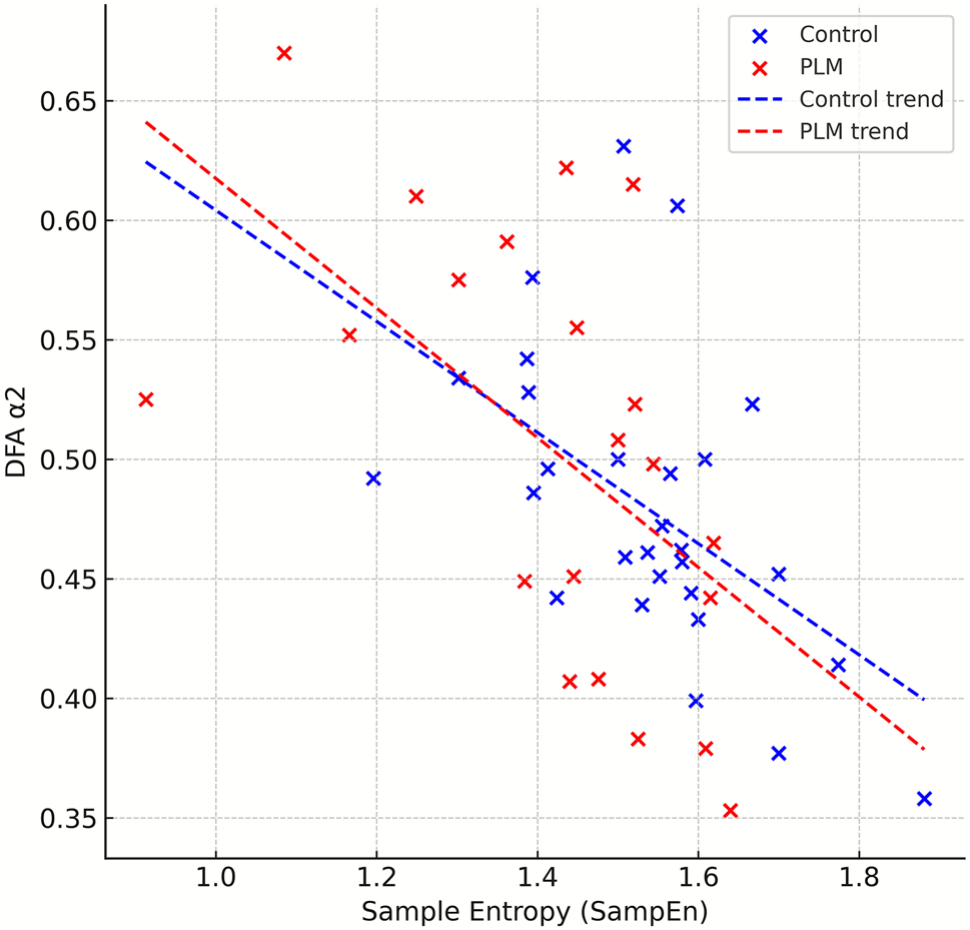
Scatter plot of sample entropy (SampEn) versus detrended fluctuation analysis (DFA) α2 during whole-night analysis

In the PLM group, both SDNN and RMSSD values were significantly higher compared to the control group. Additionally, LF power was elevated, while approximate entropy (ApEn) and sample entropy (SampEn) were significantly reduced (Fig. 1). The Stress Index was slightly lower in the PLM group. No significant differences were observed between the groups in LF/HF ratio or other HRV parameters. Likewise, there were no significant group differences in the sympathetic nervous system (SNS) and parasympathetic nervous system (PNS) indices (Table 2).

**Table 2:** Whole-night HRV results

Parameter	Control (mean ± SD)	PLM (mean ± SD)	Test	*p* value/Cohen *d* (95% CI)
Mean RR (ms)	905.64 ± 96.62	872.43 ± 101.36	Mann–Whitney *U*	0.1247/*d* = − 0.34 (− 0.84–0.17)
Mean HR (bpm)	66.96 ± 6.81	69.57 ± 7.57	Mann–Whitney *U*	0.1265/*d* = 0.36 (− 0.15–0.86)
SDNN (ms)	39.46 ± 8.81	53.24 ± 22.74	*t* test	0.0144/*d* = 0.85 (0.30–1.38)
RMSSD (ms)	37.64 ± 8.83	52.31 ± 25.94	*t* test	0.0205/*d* = 0.81 (0.25–1.35)
pNN50 (%)	12.70 ± 8.12	19.59 ± 15.01	*t* test	0.0668/*d* = 0.60 (0.08–1.10)
HRV Triangular Index	8.63 ± 2.42	10.25 ± 4.31	*t* test	0.1336/*d* = 0.48 (− 0.04–0.99)
Stress Index	6.17 ± 1.23	5.41 ± 1.34	*t* test	0.0488/*d* = − 0.59 (− 1.10 to − 0.07)
Total power (ms^2^)	1354.46 ± 565.90	2557.95 ± 2325.22	Mann–Whitney *U*	0.0738/*d* = 0.76 (0.21–1.30)
VLF power (ms^2^)	183.11 ± 129.37	309.14 ± 444.57	Mann–Whitney *U*	0.3028/*d* = 0.41 (− 0.10–0.91)
LF power (ms^2^)	674.18 ± 343.73	1373.71 ± 1371.80	Mann–Whitney *U*	0.0393/*d* = 0.75 (0.18–1.28)
HF power (ms^2^)	496.61 ± 206.39	873.76 ± 676.05	Mann–Whitney *U*	0.0976/*d* = 0.81 (0.25–1.35)
LF/HF	1.49 ± 0.76	1.82 ± 1.04	Mann–Whitney *U*	0.2067/*d* = 0.37 (− 0.14–0.87)
SD1	26.60 ± 6.24	37.00 ± 18.35	*t* test	0.0203/*d* = 0.81 (0.26–1.33)
SD2	48.20 ± 11.23	64.84 ± 28.19	*t* test	0.0170/*d* = 0.82 (0.29–1.36)
SD2/SD1	1.84 ± 0.34	1.87 ± 0.43	*t* test	0.8082/*d* = 0.07 (− 0.45–0.58)
Approximate entropy (ApEn)	1.40 ± 0.09	1.33 ± 0.12	Mann–Whitney *U*	0.0276/*d* = − 0.70 (− 1.22 to − 0.18)
Sample entropy (SampEn)	1.54 ± 0.14	1.42 ± 0.19	Mann–Whitney *U*	0.0423/*d* = − 0.72 (− 1.25 to − 0.20)
DFA alpha1	0.99 ± 0.15	1.06 ± 0.13	*t* test	0.1305/*d* = 0.43 (− 0.08–0.93)
DFA alpha2	0.48 ± 0.06	0.50 ± 0.09	*t* test	0.3021/*d* = 0.32 (− 0.18–0.82)
PNS index	− 0.17 ± 0.52	0.08 ± 0.97	*t* test	0.2959/*d* = 0.33 (− 0.17–0.83)
SNS index	− 0.42 ± 0.55	− 0.40 ± 0.60	Mann–Whitney *U*	0.5511/*d* = 0.03 (− 0.48–0.53)

*SD* standard deviation, *M* median

### HRV analysis during N2 sleep stage

**Fig. 2:**
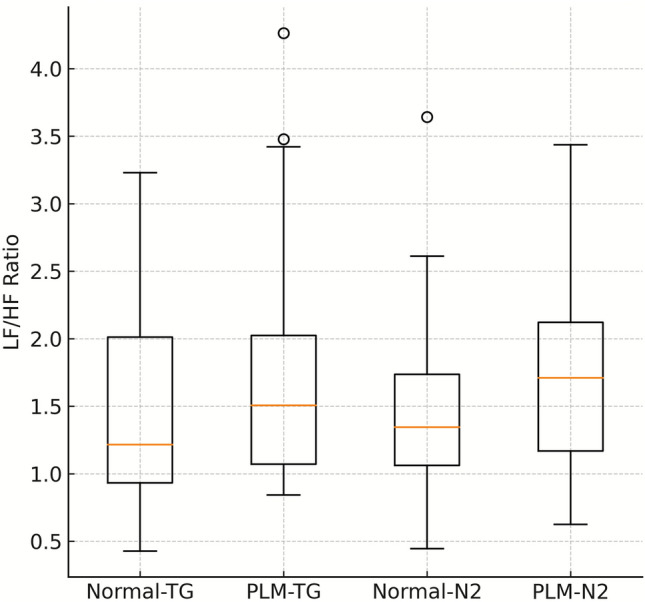
Comparison of LF/HF ratio between PLM and control groups across whole-night and N2 segments

During N2 sleep, the PLM group exhibited significantly higher SDNN, SD2, and very low frequency (VLF) power compared to the control group. Among the non-linear parameters, approximate entropy (ApEn) was lower, while detrended fluctuation analysis α1 (DFA α1) was significantly higher in the PLM group. Although the LF/HF ratio tended to be higher in the PLM group, this difference did not reach statistical significance (Fig. 2). No significant differences were observed between the groups in the sympathetic nervous system (SNS) and parasympathetic nervous system (PNS) indices (Table 3).

**Table 3:** N2 HRV results

Parameter	Control (mean ± SD)	PLM (mean ± SD)	Test	*p* value/Cohen *d* (95% CI)
Mean RR (ms)	934.57 ± 87.73	899.14 ± 131.76	*t* test	0.2936/*d* = − 0.33 (− 0.83–0.17)
Mean HR (bpm)	64.79 ± 6.07	68.00 ± 9.67	*t* test	0.1904/*d* = 0.41 (− 0.09–0.91)
SDNN (ms)	37.29 ± 11.13	53.52 ± 28.26	*t* test	0.0198/*d* = 0.80 (0.27–1.34)
RMSSD (ms)	39.27 ± 14.72	50.43 ± 30.27	Mann–Whitney *U*	0.4249/*d* = 0.49 (− 0.02–0.99)
pNN50 (%)	15.85 ± 13.26	20.46 ± 19.73	Mann–Whitney *U*	0.7777/*d* = 0.28 (− 0.22–0.78)
HRV Triangular Index	9.74 ± 3.26	11.28 ± 5.58	*t* test	0.2786/*d* = 0.35 (− 0.15–0.85)
Stress Index	8.70 ± 2.57	7.86 ± 4.48	Mann–Whitney *U*	0.0347/*d* = − 0.24 (− 0.75–0.27)
Total power (ms^2^)	1419.00 ± 839.75	2976.33 ± 2852.50	Mann–Whitney *U*	0.0997/*d* = 0.79 (0.25–1.33)
VLF power (ms^2^)	119.39 ± 96.92	301.48 ± 360.29	Mann–Whitney *U*	0.0166/*d* = 0.74 (0.22–1.28)
LF power (ms^2^)	692.61 ± 505.80	1665.33 ± 1823.34	Mann–Whitney *U*	0.0537/*d* = 0.78 (0.25–1.32)
HF power (ms^2^)	595.68 ± 390.01	1007.62 ± 948.41	Mann–Whitney *U*	0.5512/*d* = 0.60 (0.10–1.10)
LF/HF	1.45 ± 1.12	2.24 ± 1.59	Mann–Whitney *U*	0.0956/*d* = 0.59 (0.06–1.12)
SD1	27.78 ± 10.39	35.67 ± 21.40	Mann–Whitney *U*	0.4367/*d* = 0.49 (− 0.02–1.00)
SD2	44.28 ± 14.12	66.51 ± 34.77	*t* test	0.0106/*d* = 0.89 (0.35–1.42)
SD2/SD1	1.67 ± 0.41	1.98 ± 0.44	*t* test	0.0161/*d* = 0.73 (0.19–1.26)
Approximate entropy (ApEn)	1.43 ± 0.13	1.35 ± 0.17	Mann–Whitney *U*	0.0413/*d* = − 0.55 (− 1.08 to − 0.02)
Sample entropy (SampEn)	1.54 ± 0.23	1.49 ± 0.27	Mann–Whitney *U*	0.4486/*d* = − 0.22 (**− **0.73–0.28)
DFA alpha1	0.96 ± 0.24	1.15 ± 0.21	Mann–Whitney *U*	0.0084/*d* = 0.86 (0.31–1.39)
DFA alpha2	0.39 ± 0.11	0.45 ± 0.13	*t* test	0.0776/*d* = 0.54 (0.02–1.06)
PNS index	0.06 ± 0.62	0.13 ± 1.18	*t* test	0.7940/*d* = 0.08 (− 0.42–0.58)
SNS index	− 0.22 ± 0.58	− 0.10 ± 1.05	*t* test	0.6377/*d* = 0.15 (− 0.35–0.65)

*SD* standard deviation, *M* median

Overall, whole-night analyses revealed more widespread alterations across multiple HRV domains, whereas stage N2 highlighted more specific patterns, particularly an increase in DFA α1 and SD2.

### PLM characteristics

The number and index of PLMs during N2 sleep, as well as PLM-associated arousal parameters, were significantly higher in the PLM group compared to controls (Table 4).

**Table 4:** PLM characteristics

Parameter	Control (mean ± SD)	PLM (mean ± SD)	Test	*p* value/Cohen *d* (95% CI)
N2 PLMS count	0.57 ± 1.85	36.29 ± 33.26	Mann–Whitney *U*	*p* < 0.001/*d* = 1.64 (0.99–2.30)
N2 PLMS index (/h)	0.50 ± 1.97	43.55 ± 35.22	Mann–Whitney *U*	*p* < 0.001/*d* = 1.87 (1.19–2.55)
N2 PLMS–arousal count	0.00 ± 0.00	0.52 ± 1.03	Mann–Whitney *U*	0.0075/*d* = 0.77 (0.19–1.36)
N2 PLMS–arousal index (/h)	0.00 ± 0.00	0.57 ± 1.33	Mann–Whitney *U*	0.0075/*d* = 0.66 (0.08–1.24)

## Discussion

This observational, hypothesis-generating study demonstrates that patients with PLMS exhibit widespread autonomic alterations throughout the entire night, as well as distinct differences in certain parameters during stage N2 non-rapid eye movement (NREM) sleep. In whole-night analyses, the PLM group showed increased standard deviation of normal-to-normal intervals (SDNN), root mean square of successive differences (RMSSD), and low-frequency (LF) power; reduced entropy measures, including approximate entropy and sample entropy; and lower Stress Index values. During stage N2, SDNN, Poincaré long-axis standard deviation and detrended fluctuation analysis alpha-1 were elevated, approximate entropy was reduced, and very-low-frequency (VLF) power was increased; the low-frequency/high-frequency (LF/HF) ratio showed a trend toward elevation. No significant group differences were observed in high-frequency (HF) power or in the composite sympathetic nervous system index and parasympathetic nervous system index. Furthermore, the increased PLMS burden and arousal-related parameters observed in N2 support the notion of additional autonomic effects specific to this stage. Graphical analyses illustrated reduced sample entropy and a trend toward higher detrended fluctuation analysis alpha-2 in the PLM group, suggesting a possible disruption of fractal organization in PLMS.

Time-domain parameters are statistical indices derived from the series of NN (normal–normal) intervals between consecutive heartbeats and are widely accepted as key markers of autonomic nervous system activity [6, 12]. Several measures have been described in this category, including the standard deviation of normal-to-normal intervals (SDNN), the root mean square of successive differences (RMSSD), pNN50, and the HRV Triangular Index. In the present work, we concentrated on the two most frequently applied markers—SDNN and RMSSD—to characterize autonomic changes associated with PLMS. SDNN is regarded as the most fundamental indicator of overall HRV, reflecting contributions from both sympathetic and parasympathetic branches, and is therefore considered a measure of global autonomic adaptability [6]. Earlier studies generally reported reduced SDNN values in patients with PLMS [3, 14]. In contrast, Sforza and colleagues (2005, 2019) [9, 15] documented higher SDNN during PLM episodes, suggesting that the condition may give rise to broad autonomic oscillations throughout sleep. Our findings are consistent with this view, as we also observed significantly elevated SDNN levels in the PLM group across both whole-night and N2 analyses. By comparison, RMSSD mainly reflects parasympathetic (vagal) activity and shows strong correspondence with the high-frequency component of HRV [6]. Evidence regarding RMSSD in PLMS remains inconsistent, with some investigations reporting no significant differences [3, 14]. In our sample, RMSSD increased significantly only in the whole-night analysis, while no changes were detected during stage N2. Overall, the persistent elevation of SDNN in both the whole-night and N2 segments suggests that PLMS may drive generalized autonomic variability. Meanwhile, the selective increase of RMSSD in whole-night recordings implies that parasympathetic regulation could be unevenly attenuated depending on the sleep stage. These observations point to SDNN as a more reliable marker for evaluating periodic-limb-movement–related autonomic variability. Given the established association between reduced HRV and cardiovascular morbidity, the observed SDNN elevation in PLMS may indicate compensatory autonomic fluctuations rather than overt sympathetic excess.

Frequency-domain analysis examines the spectral distribution of heart rate intervals, offering information on the relative contributions of sympathetic and parasympathetic modulation [6, 12]. LF power, while shaped by inputs from both systems, is most often interpreted as a marker of sympathetic predominance. Several prior investigations have described elevated LF power in PLMS, supporting the view of sympathetic overactivity [8, 13, 16]. In our dataset, LF power was significantly higher in the whole-night recordings, whereas during stage N2 it only showed a nonsignificant upward trend. HF power, in contrast, primarily reflects parasympathetic influence and is regarded as the most consistent indicator of vagal modulation linked to respiration [6]. In our cohort, HF values did not differ between groups. The LF/HF ratio has traditionally been used to estimate sympathovagal balance, though more recent reports caution that this index alone is insufficient for reliable interpretation [6, 17]. In our analysis, the PLM group exhibited a tendency toward increased LF/HF during N2, but the difference did not reach significance. This partial dissociation between elevated LF and unchanged HF values may reflect the time-dependent autonomic co-activation model described by Małkiewicz et al. (2024, 2025), rather than a purely sympathetic predominance [4, 10]. Taken together, the results suggest that PLMS is associated with heightened sympathetic drive (↑LF), intact parasympathetic function (↔ HF), and a possible shift toward sympathovagal imbalance (↑LF/HF trend).

Nonlinear HRV analysis, extending beyond conventional time- and frequency-domain indices, evaluates the complexity and fractal behavior of cardiac dynamics [7, 18–20]. In our study, entropy measures—approximate entropy and sample entropy—were lower in the PLM group, indicating a shift toward more regular and predictable heart rate fluctuations. Approximate entropy was consistently reduced in both whole-night and N2 analyses, whereas sample entropy declined significantly only in the whole-night segment. These findings imply that autonomic regulation in PLMS is characterized by diminished physiological complexity, potentially limiting cardiovascular adaptability. Examination of detrended fluctuation analysis metrics further demonstrated that detrended fluctuation analysis alpha-1, which reflects short-term fractal characteristics, was significantly elevated in the PLM group during stage N2. By contrast, detrended fluctuation analysis alpha-2, associated with long-term correlations, did not differ significantly between groups, although values in the PLM group tended to be higher [21–25]. Overall, these results suggest that PLMS—particularly in stage N2—may involve increased deterministic organization and altered short-term sympathovagal dynamics.

The Stress Index is a HRV parameter derived from the distribution of RR intervals and is commonly interpreted as an indicator of sympathetic predominance and overall stress burden. Higher Stress Index values reflect enhanced sympathetic drive, whereas lower values are generally associated with parasympathetic influence [6]. In our analysis, the Stress Index was consistently reduced in the PLM group in both whole-night and N2 segments. To our knowledge, this is the first study to describe Stress Index alterations in an adult population with PLMS. Notably, while conventional HRV measures suggested sympathetic activation, the parallel decrease in the Stress Index indicates an atypical autonomic pattern in PLMS. Such a profile may reflect sympathovagal co-activation, particularly during stage N2. Taken together, the lower Stress Index in our cohort suggests an autonomic phenotype that diverges from the classic model of sympathetic overactivity. This novel observation is hypothesis-generating and warrants further investigation.

Our study demonstrates that PLMS not only disrupt sleep microstructure but also exert distinct effects on the autonomic nervous system. The findings indicate that PLMS lead to widespread autonomic fluctuations throughout the night, whereas stage N2 is characterized by stage-specific contributions, namely increased SDNN, Poincaré long-axis standard deviation, and detrended fluctuation analysis alpha-1, along with reduced approximate entropy. This pattern can be summarized as increased physiological variability accompanied by reduced autonomic complexity.

Previous studies have shown that PLMS is associated with heightened sympathetic activation, which may contribute to an increased long-term risk of hypertension, arrhythmia, and stroke [3, 5, 8, 13, 14, 16, 26]. Our study identifies unique autonomic patterns that may be linked to these clinical outcomes. While our results are consistent with the existing literature, the reduction in entropy and the elevation of detrended fluctuation analysis alpha-1 observed in nonlinear parameters suggest that PLMS may influence autonomic regulation not only through sympathetic activation but also via a loss of complexity and disruption of fractal dynamics [21–25]. This pattern indicates that PLMS may represent an atypical autonomic phenotype distinct from the classical markers of sympathetic activation and constitutes the novel contribution of our study. Such findings may aid in the development of new biomarkers for cardiovascular risk assessment in PLMS. Nevertheless, our results are hypothesis-generating, and larger prospective studies are needed to confirm these associations.

The Stress Index, evaluated here for the first time in an adult PLMS cohort, did not align with the expected sympathetic activation patterns. Instead, consistently lower Stress Index values indicated an unusual autonomic phenotype in PLMS. This observation may serve as a novel, hypothesis-generating finding that warrants further investigation in future research.

## Limitations

Our study has several limitations. First, the sample size was relatively small, which may limit the reliability of nonlinear HRV parameters. Second, although an AHI < 10 criterion was applied, the potential influence of residual respiratory events on HRV could not be completely excluded. Third, only single-night PSG recordings were analyzed, and night-to-night variability was not assessed. Because the study was retrospective, several potentially relevant physiological variables—such as menstrual or hormonal status in female participants—could not be controlled. Furthermore, HRV reflects integrated effects of autonomic, respiratory, and micro-arousal processes; therefore, the present findings should be interpreted as associative rather than causal. Finally, the duration of individual PLMS events was not available for analysis, precluding evaluation of possible duration-dependent autonomic effects reported in recent literature. Considering these limitations, our findings should be regarded as hypothesis-generating, and larger, multi-night, prospective studies are needed to confirm these results.

## Conclusions

This study demonstrates that PLMS exerts significant effects on the autonomic nervous system throughout the night and, in addition, reveals distinct alterations in certain parameters during stage N2. The findings suggest that PLMS may lead to increased cardiac variability accompanied by reduced complexity, pointing toward an atypical autonomic phenotype. These results should be considered hypothesis-generating and require confirmation in larger, prospective cohorts.

## Data Availability

The data presented in this study are available from the corresponding author upon reasonable request.
